# Mutation of *foxl1* Results in Reduced Cartilage Markers in a Zebrafish Model of Otosclerosis

**DOI:** 10.3390/genes13071107

**Published:** 2022-06-21

**Authors:** Alexia Hawkey-Noble, Justin A. Pater, Roshni Kollipara, Meriel Fitzgerald, Alexandre S. Maekawa, Christopher S. Kovacs, Terry-Lynn Young, Curtis R. French

**Affiliations:** Division of Biomedical Sciences, Faculty of Medicine, Memorial University of Newfoundland, St. John’s, NL A1B 3V6, Canada; amhn83@mun.ca (A.H.-N.); justin.pater@gmail.com (J.A.P.); rskk10@mun.ca (R.K.); mjf637@mun.ca (M.F.); asmaekawa@mun.ca (A.S.M.); ckovacs@mun.ca (C.S.K.); tlyoung@mun.ca (T.-L.Y.)

**Keywords:** *foxl1*, *foxc1*, zebrafish, otosclerosis, collagen, osteoporosis, iron binding, bone mineral density

## Abstract

Bone diseases such as otosclerosis (conductive hearing loss) and osteoporosis (low bone mineral density) can result from the abnormal expression of genes that regulate cartilage and bone development. The forkhead box transcription factor *FOXL1* has been identified as the causative gene in a family with autosomal dominant otosclerosis and has been reported as a candidate gene in GWAS meta-analyses for osteoporosis. This potentially indicates a novel role for *foxl1* in chondrogenesis, osteogenesis, and bone remodelling. We created a *foxl1* mutant zebrafish strain as a model for otosclerosis and osteoporosis and examined jaw bones that are homologous to the mammalian middle ear bones, and mineralization of the axial skeleton. We demonstrate that *foxl1* regulates the expression of collagen genes such as *collagen type 1 alpha 1a* and *collagen type 11 alpha 2*, and results in a delay in jawbone mineralization, while the axial skeleton remains unchanged. *foxl1* may also act with other forkhead genes such as *foxc1a*, as loss of *foxl1* in a *foxc1a* mutant background increases the severity of jaw calcification phenotypes when compared to each mutant alone. Our zebrafish model demonstrates atypical cartilage formation and mineralization in the zebrafish craniofacial skeleton in *foxl1* mutants and demonstrates that aberrant collagen expression may underlie the development of otosclerosis.

## 1. Introduction

Vertebrate skeletal development requires extracellular signalling cues and transcription factor expression to direct the development of cartilage, bone, and other connective tissues [1,2,3,4]. In vertebrates, cranial neural crest cells (NCCs) derived from a specialized population within the first, second, and third pharyngeal arches are responsible for developing into the progenitor cells of the craniofacial skeleton, while the paraxial mesoderm condenses into epithelial somites that give rise to the sclerotome from which the axial skeleton develops [3,5]. This cellular commitment is induced by external paracrine factors leading to *pax1* and *scleraxis* expression [1,6], followed by subsequent expression of transcription factors (TFs), such as those of the *SOX* gene family in skeletal progenitors that aid in chondrogenesis and osteogenesis [7,8]. Many other well-known signaling cascades such as Hedgehog (Hh) [9,10,11,12,13,14,15], Fibroblast growth factor (Fgf) [16,17], Jagged-Notch, and Bone Morphogenic Protein (Bmp) signalling [16,18,19,20] are also required to direct progenitor cells into their specialized skeletal fates. Mutation of genes in these pathways can result in developmental defects in cartilage and bone formation/remodelling and can result in bone diseases such as otosclerosis (conductive hearing loss from abnormal middle ear bone growth) or osteoporosis (low bone density) [10,16,17,18,20].

Forkhead box transcription factors (FOX) have been previously shown to be crucial in craniofacial patterning as well as in the differentiation of chondrocytes and osteoblasts in humans, mice, and zebrafish [17,21,22,23,24,25,26]. FOX TFs contain highly conserved DNA-binding domains consisting of 110 amino acids, and may enhance or repress downstream gene expression by binding to forkhead consensus sequences in regulatory regions of the DNA [27,28]. Several FOX genes, such as those in classes C and F [25], have been studied regarding craniofacial and axial skeletal development. This prior work suggests an inherent FOX expression map resulting from the partial overlap of FOX genes to create distinct boundaries that position bone and cartilage fields in the head during development. Genes such as *Foxc1* [mouse, (*foxc1a* in zebrafish)] and *Foxc2* [mouse (*foxc1b* in zebrafish)] are required for chondrocyte and osteoblast differentiation, contributing to much of the expression map necessary for dermal bone formation (derived from intramembranous and endochondral ossification) in the upper and lower facial cartilages [23,25,29,30,31]. As such, they are associated with diseases such as Axenfeld–Rieger syndrome, which often presents with craniofacial abnormalities and hearing loss [21,32,33,34].

Recently, a mutation in the forkhead gene (*FOXL1*) was identified as the causative gene of autosomal dominant otosclerosis in a large Newfoundland family [35], and previous studies have associated this gene with osteoporosis [36,37,38,39]. In animal models, *foxl1* is expressed in the paraxial mesoderm and NCCs of pharyngeal arches, and is a downstream target of both BMP and Hh signalling [40,41], yet few studies have examined *foxl1’s* role in bone development and remodelling of the craniofacial and axial skeleton. Given its association with two bone remodelling disorders, further study into its role in regulating skeletal development and remodelling in conjunction with other forkhead transcription factors is warranted. 

Herein, we utilize a zebrafish model to determine the role of *foxl1* in cartilage and bone development in the embryonic head and axial skeleton to further understand its role in disease pathogenesis. We also utilize two previously studied zebrafish forkhead mutants (*foxc1a* and *foxc1b*) [42,43] in conjunction with a new *foxl1* mutant line to examine the importance of *foxl1* in the FOX map. As key cartilage/bone developmental and remodelling pathways are conserved between teleosts and mammals [4,10,16,31,44,45,46,47,48,49,50], as well as aspects of the zebrafish jaw being homologous to the middle ear bones of mammals [49,51,52,53], zebrafish make an excellent model for the study of otosclerosis and osteoporosis [54,55,56,57,58]. We find that while *foxl1* regulates the expression of known markers of chondro/osteogenesis, CRISPR-induced mutation of *foxl1* in zebrafish only results in a delay in the formation of craniofacial cartilages and subsequent calcification, with no apparent effects on axial skeletal development. As loss of *foxl1* is insufficient to overtly alter skeletal development, we assessed skeletal patterning in *foxl1* mutants crossed onto *foxc1a* or *foxc1b* mutant backgrounds, which overlap in expression domains with *foxl1*. We find that *foxl1/foxc1a* double mutants exhibit an exacerbated phenotype with more severe calcification defects in the craniofacial skeleton when compared to either mutant alone, and that *foxc1a* and *foxl1* share gene targets involved in skeletal development. Thus, we propose that *foxl1* is dispensable for overall craniofacial and axial skeleton development but may act as a modifier locus for *foxc1a*. 

## 2. Materials and Methods

### 2.1. Zebrafish Husbandry

As per Kimmel et al. [59], both wildtype (strain AB) and mutant zebrafish were reared under standard conditions and staged in terms of hours post fertilization (hpf) or days post fertilization (dpf), as described. All experiments were performed following the regulations and procedures outlined by Memorial University of Newfoundland’s Animal Care Committee and the Canadian Council on Animal Care. To prevent pigmentation and ensure optic clarity, all embryos 24 hpf and older were subject to 0.003% 1-phenyl 2-thiourea (PTU; Sigma-Aldrich, St. Louis, MO, USA). A total of 0.168 mg/mL tricaine was used to anesthetize embryos 48 hpf and older during experiments or prior to fixation with 4% paraformaldehyde (PFA; Sigma-Aldrich, St. Louis, MO, USA). 

### 2.2. Zebrafish Strains Used in This Study

CRISPR-Cas9 sgRNAs were designed to target zebrafish *foxl1* zebrafish. Two sgRNAs were designed to bind before the forkhead DNA binding domain (Supplemental Table S1) and were co-injected inducing a 52 bp deletion and shift in the open reading frame before this critical region (Supplemental Figure S1). We have designated this allele *foxl1^n1001^*. Experiments were performed by crossing heterozygous fish to produce homozygous embryos and wildtype (WT) sibling controls, or by crossing homozygous fish and WT (AB strain fish) for comparison. Previously generated *foxc1a^ua101^* and *foxc1b^ua1018^* alleles [42,43] were utilized for single and double mutant analysis. 

### 2.3. Alcian Blue and Alizarin Red Staining

Larvae were stained at 6 and 10 dpf using an acid-free alcian blue and alizarin red double stain as previously reported [60]. Up to 50 larvae were collected in a 1.5 mL microcentrifuge tube and fixed for 2 h in 4% *w*/*v* paraformaldehyde in phosphate buffered saline pH 7.4 with gentle shaking at room temperature. After washing with 1 mL 50% *v*/*v* ethanol, larvae were gently shaken in 1 mL 50% ethanol for 10 min. The ethanol solution was removed and replaced with 1 mL of alcian blue (0.01% *w*/*v* alcian blue 8 GX, 100 mmol·L^−1^ MgCl_2_, 70% *v*/*v* ethanol) and 25 µL of alizarin red (5 mg·mL^−1^ alizarin red in ultrapure water). Larvae were incubated in the staining solution for 1 h at room temperature with gentle rocking. The stain solution was removed, and larvae were washed with 1 mL ultrapure water, followed by addition of 1 mL bleaching solution (1.5% *v*/*v* H2O2, 1% *w*/*v* KOH in ultrapure water). Larvae were incubated in bleaching solution for 20 min at room temperature with the microcentrifuge tubes uncapped. After removing the bleaching solution, a solution of 20% *w*/*v* glycerol and 0.25% *w*/*v* KOH was added. Larvae were gently rocked at room temperature for 30 min, before the solution was replaced with 50% *w*/*v* glycerol and 0.25% *w*/*v* KOH. Larvae were incubated overnight at 4 °C before being imaged and transferred to a storage solution (50% *w*/*v* glycerol and 0.1% *w*/*v* KOH), before being genotyped. For genotyping, individual larvae were washed twice with 100 µL PBSTw (phosphate buffered saline 0.1% *w*/*v* Tween 20) and once with 100 µL PCR-clean water. Larvae were then boiled in 50 µL 50 mmol·L^−1^ NaOH for 20 min at 95 °C and cooled to room temperature before addition of 5 µL 1 mol·L^−1^ Tris-HCl pH 8. The resulting solution was used as the template for PCR.

### 2.4. Whole Mount In Situ Hybridizations

In accordance with Thisse and Thisse [61], an in situ hybridization probe (*foxl1*) was synthesized from a pooling of whole-body RNA. PCR amplicons for probe synthesis were generated using the One Step Superscript IV RT-PCR Kit (Invitrogen). Antisense probes labelled with DIG (Roche) were constructed using an incorporated T7 RNA polymerase promoter (added at the 5′ end of the antisense primer). Proteinase K was used to permeabilize older embryos before incubation with probe: 3 min for 24 hpf, 18 min (48 hpf). DIG labelled probes were detected using alkaline phosphatase coupled anti-DIG FAB fragments, with subsequent coloration via nitro-blue tetrazolium (NBT; Roche) and 5-bromo-4-chloro-3-indolyl phosphate (BICP; Roche). Primer sequences are listed in Supplemental Table S1. 

### 2.5. Calcein Staining

Live embryos previously growing in 0.003% PTU embryo media at ages 6 and 10 dpf were incubated in a 0.2% (*w*/*v*) calcein solution, pH 7.5 (Sigma-Aldrich, St. Louis, MO, USA), for 12 min followed by three 5-min washes in 0.003% PTU embryo media. Embryos were subsequently anesthetized in tricaine (see Zebrafish Husbandry) and mounted in 6% methylcellulose for imaging. Images were collected in the dark using a Nikon SMZ18 microscope equipped with a long-pass green filter (excitation 480 ± 40 nm; emission 510 nm). Significance testing for delayed craniofacial cartilage and bone at 6 dpf was calculated using Fisher’s exact test. Delay of cartilage development and bone calcification was characterized by decreased staining intensity, shape, and size of area (in the case of primary ossification centres), and the presence or lack thereof in comparison to their WT siblings in their respective experimental groups. 

### 2.6. TaqMan Real-Time Quantitative PCR

Total RNA was isolated using Trizol (Invitrogen) at 6 and 10 dpf from WT sibling controls and *foxl1^-/-^* mutants, as well as at 6 dpf for *foxc1a^-/-^* and *foxc1b^-/-^* mutants and WT siblings as per Peterson and Freeman [62]. cDNA was then generated using the High-Capacity cDNA Reverse-Transcription Kit (Applied Biosystems). A total of 50–100 ng of cDNA was used in each reaction. Two biological replicates (containing a pool of 20 embryos) with three technical replicates were used with each probe and normalized to expression levels of the TATA-box binding protein (*tbp*) housekeeping gene. RT-qPCR runs were completed on an Applied Biosystems 7500 Real-Time PCR System or a ViiA 7 system. Data were analyzed using the ΔΔCT method [63,64]. Data bars are given as means ± standard error of the mean (SEM) with significance testing (*p*-values) calculated using a two-tailed t-test. Previously validated TaqMan assays were purchased from Thermo Fisher as follows: *matn1* (Dr_03092841), *col1a1a* (Dr_03150834), *col1a1b* (Dr03074863), *col11a2* (Dr03085627), *hbae3* (Dr03125483), *hpx* (Dr03430535), and *sp7* (Dr03133254). 

### 2.7. DXA Scanning

Whole-body BMD assessment was performed using DXA scanning and was completed as per Green et al. [64] using a PIXImus Scanner (GE/Lunar; Madison, WI, USA) that was calibrated daily with a standard phantom. Data bars are given as means ± standard error of the mean (SEM) with significance testing (*p*-values) calculated using a one-way ANOVA to compare WT, *foxl1* heterozygotes, and homozygotes, and a two-tailed *t*-test to compare WT to *foxl1^+/-^* and *foxc1b^+/-^* mutants. 

## 3. Results

### 3.1. foxl1 Mutant Generation

A CRISPR-Cas9 induced mutant zebrafish strain was generated for *foxl1*, creating a 52 base-pair deletion in the lone *foxl1* exon. Although not degraded through the nonsense mediated decay pathway due to the gene containing a single exon, the predicted protein was expected to have a shift in the reading frame that would disrupt the forkhead DNA binding domain with a premature termination codon (PTC) after amino acid 148, removing over half the protein sequence (Supplemental Figure S1). Homozygous mutants were viable and fertile, with heterozygotes and homozygotes produced at expected Mendelian frequencies. 

### 3.2. Craniofacial Cartilage Formation and Calcification in foxl1 Mutants

**Figure 1:**
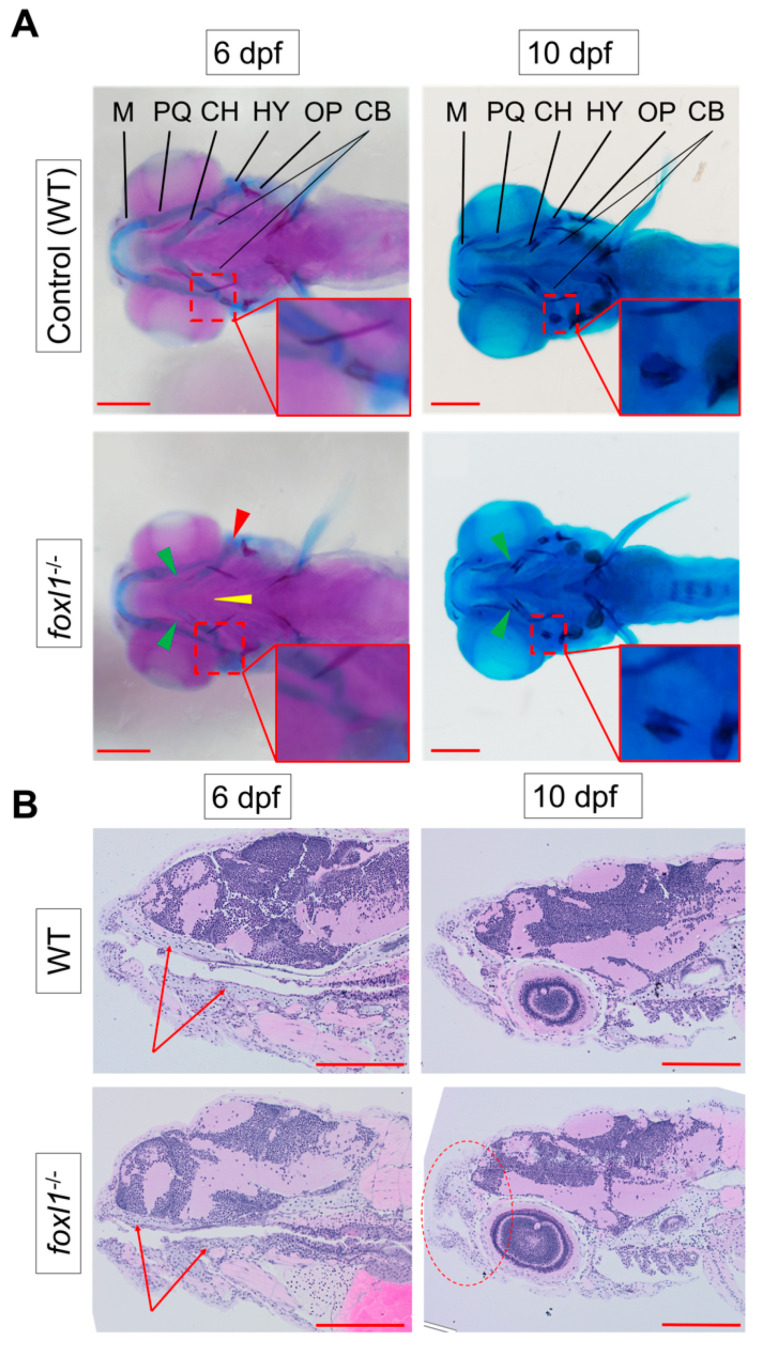
Reduction in cartilage in *foxl1* mutants. (**A**) Alcian blue (staining cartilage) of WT and *foxl1^-/-^* (6 dpf *n* = 24, 10 dpf *n* = 20) embryos, indicating a delay in ceratohyal cartilages at 6 dpf (green arrowhead), but a recovery begins by 10 dpf. Alizarin red indicates a delay in the ossification of the hyomandibula (red arrowhead and inserts) in *foxl1* mutants at 6 dpf, which also begins to normalize by 10 dpf. (**B**) Longitudinal sections through jaw, demonstrating a reduced presence of cartilage formation in the upper and lower jaw as indicated by the red arrows at 6 dpf, along with a shortened and round jaw structure that progresses by 10 dpf (red circle). PQ, palatoquadrate; M, Meckel; HY, hyomandibula (hyosympletic); CH, ceratohyal; CB, ceratobranchials (yellow arrowhead); OP, operculum. Zoomed-in inserts in each panel focus on the cartilage and primary centres of ossification of the hyomandibula. Scale bars are 200 µm.

Given the association of otosclerosis with aberrant cartilage formation [65,66,67,68,69], we assessed the underlying cartilage formation of the zebrafish jaw by using alcian blue staining combined with alizarin red at 6 and 10 dpf. Homozygous *foxl1* mutants exhibited a visible reduction in cartilage (alcian blue) staining of the ceratohyal (Figure 1A, green arrowheads), ceratobranchials (Figure 1A, yellow arrowhead), and hyomandibular cartilages at 6 dpf (Figure 1A, red arrowheads), which mostly recovered by 10 dpf. At 10 dpf, all skeletal elements were well stained, indicating a recovery in chondrogenesis (Figure 1A, 10 dpf).

Similarly, at 6 dpf, alizarin red staining for calcified bone demonstrated a reduction in calcification in *foxl1* mutants. Normal calcification of the operculum is evident, while the calcification of the hyomandibula (Figure 1A, red arrowheads) and ceratohyal cartilages (Figure 1A, 6 dpf green arrowheads) were reduced at their respective primary centres of ossification, as shown by the reduced alizarin red staining. By 10 dpf, *foxl1* mutants appear to have recovered with no major morphological differences observed at this developmental stage, but they still had less calcification at the primary centres of ossification in the ceratohyals than their WT counterparts, as indicated by the lesser staining (alizarin red, Figure 1A, 10 dpf green arrowheads). 

As we observed a reduction in cartilage formation of the jaw in *foxl1* mutants via alcian blue staining, further analysis in the form of histology with an H&E stain was used to compare jaw morphology between WT and *foxl1^-/-^* larvae at 6 and 10 dpf (Figure 1B). It was evident that the *foxl1^-/-^* mutants had reduced cartilage elements (arrows Figure 1B) and a shortened and rounded jawline that became more prominent as the embryos aged from 6 to 10 dpf (circle, Figure 1B). 

**Figure 2:**
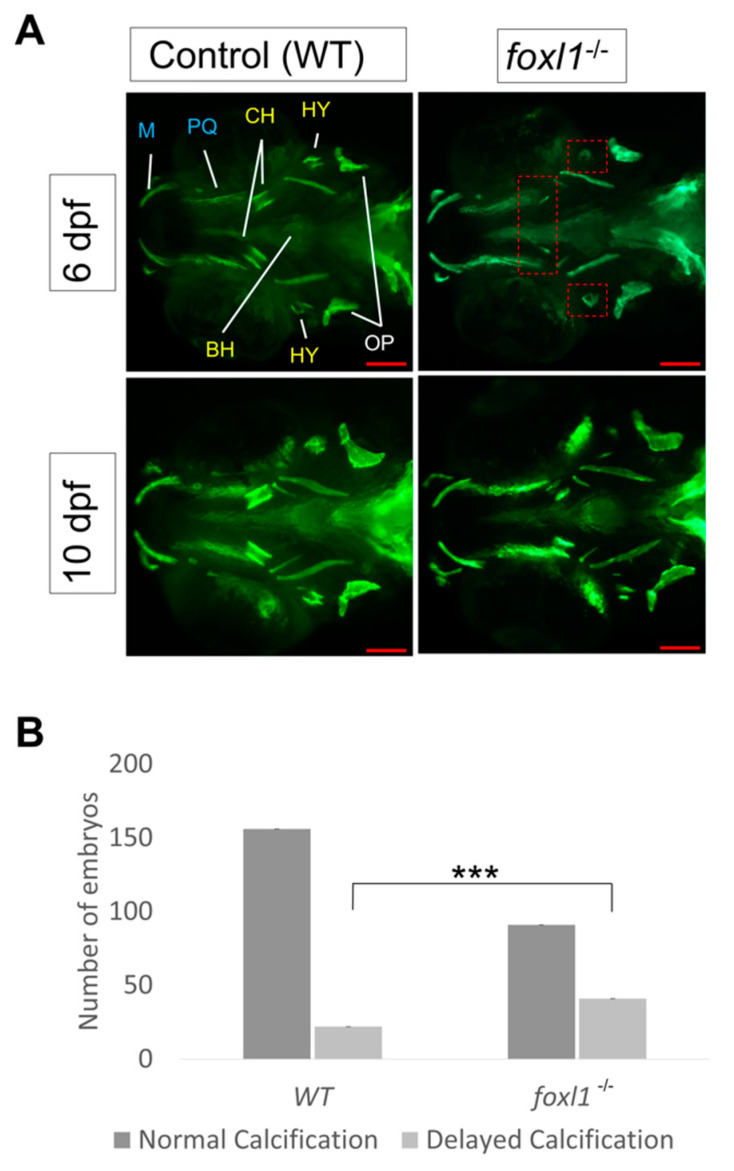
Calcein staining illustrating the impact of *foxl1* loss on craniofacial development and calcification. (**A**) Embryos of WT aged 6 and 10 dpf (*n* = 173 and 99, respectively) as well as those of *foxl1^-/-^* (*n* = 116 and 65, respectively). WT embryos at both 6 and 10 dpf exhibit normal craniofacial development and calcification of all jaw structures. *foxl1^-/-^* embryos show a delayed calcification in the ceratohyal and hyomandibula (red boxes) at 6 dpf yet appear mostly recovered by 10 dpf. (**B**) The proportion of embryos with delayed calcification in *foxl1* mutants is statistically significant (Fisher’s *** *p* = 0.0001) when compared to wildtype siblings. PQ, palatoquadrate; M, Meckel; HY, hyomandibula (hyosympletic); BH, basihyal; OP, opercula; CH, ceratohyal. Scale bars are 100 µm.

To further visualize the effect that the *foxl1* mutation has on bone formation and calcification, calcein staining was performed on live embryos at both 6 and 10 dpf. The hyomandibula and ceratohyal were examined closely as they are the homologous structures of the stapes (middle ear bone) in mammals, which is often the afflicted structure in otosclerosis and was delayed in its calcification according with alizarin red staining. At 6 dpf, fewer *foxl1* homozygous mutant embryos had complete calcification of the hyomandibula and ceratohyal bones when compared to wildtype siblings, as indicated by the reduced staining/presence of calcein (*p* = 0.0001, Figure 2A,B). Both ossification centres of the hyomandibula were observed at by 10 dpf, albeit with less intense staining, indicating a partial recovery of calcification (Figure 2A). These data agree with the alizarin red staining and indicate a delay in calcification specifically at primary centres of ossification in the developing jaw. 

### 3.3. Overlapping Expression of foxl1, foxc1a, and foxc1b in Zebrafish

**Figure 3:**
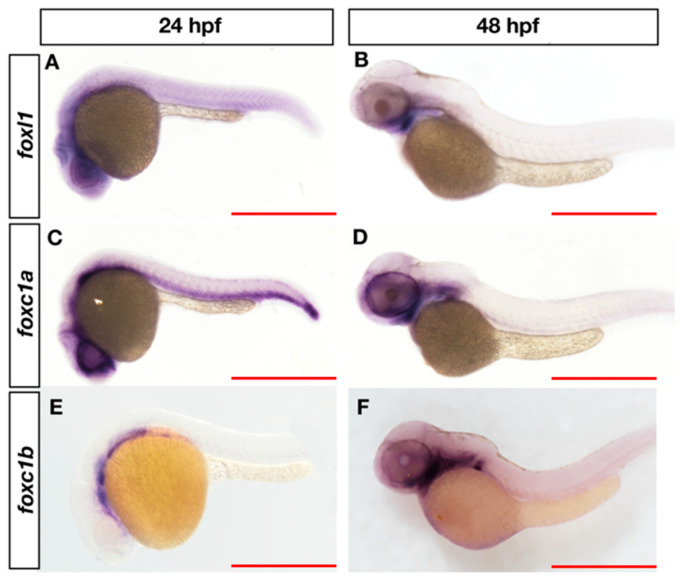
Expression of *foxl1*, *foxc1a,* and *foxc1b* in wildtype zebrafish at 24 and 48 hpf. At 24 hpf, *foxl1* is expressed at in the brain and trunk (**A**), similar to *foxc1a* (**C**), while *foxc1b* is observed in the ventral head/brain regions (**E**). At 48 hpf, all three forkhead genes are expressed in the pharyngeal arches (**B**,**D**,**F**). Scale bars are 500 µm.

Given that phenotypes in jaw development recover in *foxl1* mutants as development proceeds, we asked whether genetic redundancy could allow for overtly normal jaw development in *foxl1* mutants. We assessed the expression of other forkhead genes that can cause craniofacial and axial skeletal defects in zebrafish and humans when mutated, mainly *foxc1a* and *foxc1b* (*FOXC2*) [24,25]. All three *fox* genes are expressed in the head in a pattern consistent with NCC development at 24 hpf (Figure 3A,C,E) and in the pharyngeal arches (Figure 3B,D,F) that are predominantly responsible for giving rise to Meckel’s, palatoquadrate, ceratohyal, and hyomandibular cartilages by 48 hpf. Expression in the ventral somite domains is observed for both *foxl1* and *foxc1a* at 24 hpf.

### 3.4. foxl1 Mutation in foxc1a and foxc1b Mutant Backgrounds 

**Figure 4:**
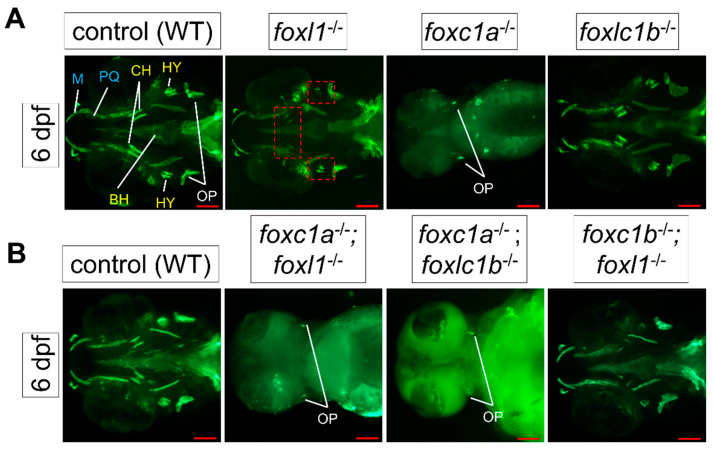
Calcein staining illustrating the impact of *foxl1, foxc1a,* and *foxc1b* lone and combined loss on craniofacial development and calcification. (**A**) *foxc1a^-/-^* embryos have a lack of bone development in all craniofacial bones resulting from the first (blue) and second (yellow) pharyngeal arches and diminished formation of the opercula bones at 6 dpf. *foxc1b^-/-^* embryos exhibit no change. *foxl1* panels are included for reference. (**B**) WT (*n* = 173); *foxc1a^-/-^*; *foxl1^-/-^* (*n* = 6); *foxc1b^-/-^*; *foxl1^-/-^* (*n* = 2); and *foxc1a^-/-^*; *foxc1b^-/-^* (*n* = 4) embryos at 6 dpf. *foxc1a^-/-^*; *foxl1^-/-^* embryos exhibit a further loss of craniofacial bone formation and calcification with increased size in the cardiac edema present, while *foxc1b^-/-^*; *foxl1^-/-^* embryos exhibit no change from their respective individual knockout models. *foxc1a^-/-^*; *foxc1b^-/-^* double mutants show a similar phenotype as *foxc1a^-/-^*; *foxl1^-/-^* embryos. Red boxes highlight areas of interest. Small red squares highlight the hyomandibula and the large rectangular box isolates the ceratohyal bone. Scale bars are 100 µm.

To assess the effect that *foxl1* mutation has in combination with other forkhead mutants, calcein staining was performed on live embryos at 6 dpf. In agreement with other studies [24,25], *foxc1b* mutants did not exhibit any morphological changes in comparison to WT embryos; however, loss of *foxc1a* in zebrafish embryos clearly resulted in the lack of development of most major craniofacial structures such as the hyomandibula, palatoquadrate, and Meckel’s composing the developing jaw (Figure 4A). *foxc1a* mutants also display hydrocephalous and cardiac/abdominal edema compared to wildtype siblings. *foxc1a* homozygous mutants die around 7 dpf.

Double *foxc1a^-/-^; foxl1^-/-^* mutants (Figure 4B) exhibited reduced bone formation, including a lack of calcification of the operculum, when compared to *foxc1a* mutants alone which exhibit mostly normal operculum calcification. Unlike *foxc1a^-/-^; foxl1^-/-^* double mutants, *foxl1^-/-^*; *foxc1b^-/-^* embryos did not exhibit an abnormal phenotype in comparison to the wildtype controls (Figure 4B). *foxc1a^-/-^*; *foxc1b^-/-^* double mutant embryos had similar phenotypes as the *foxc1a^-/-^*; *foxl1^-/-^* embryos at 6 dpf, which included further loss of bone calcification in the face (Figure 4B). 

### 3.5. Axial Skeletal Calcification in Forkhead Mutants

**Figure 5:**
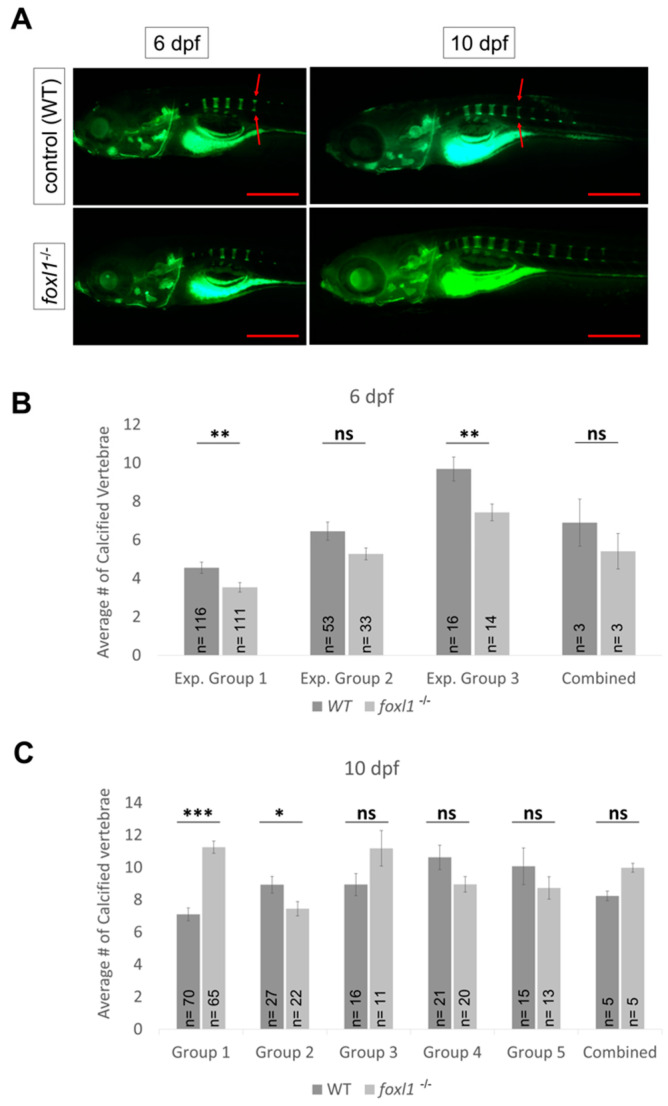
Calcein staining illustrating the impact of *foxl1* loss on axial skeletal development and calcification. (**A**) WT and *foxl1^-/-^* embryos at 6 and 10 dpf showing normal formation of the vertebrae in *foxl1*. (**B**,**C**) Experimental groups of calcein staining illustrating the variability in calcification of the zebrafish vertebrate between WT and *foxl1* homozygotes at 6 and 10 dpf, respectively (* *p* < 0.05, ** *p* < 0.01, *** *p* < 0.001), showing an increased trend in number of calcified vertebrae in *foxl1* mutants by 10 dpf. Total number of calcified vertebrae were counted by including both complete and partially calcified vertebrae [partially calcified vertebrae are indicated by disconnected primary and secondary centres of ossification (red arrows)]. Scale bars are 500 µm.

In humans, variants in *FOXL1* and *FOXC2* are associated with a reduced bone mineral density (BMD) and a risk of osteoporosis [35,36,39]. Furthermore, the co-occurrence of otosclerosis and osteoporosis has been noted [65], suggesting similar aetiologies for both bone remodelling diseases. We thus tested *foxl1*, *foxc1a*, and *foxc1b* for defects in the calcification of the zebrafish spine to determine if either gene affects calcification of the axial skeleton. Mutation of *foxl1* has little effect on the patterning and formation of the vertebrae of the skeleton at 6 and 10 dpf when compared with WT controls (Figure 5), as vertebral calcification appeared to be variable within experimental groups (Figure 5B,C). This could possibly be due to environmental changes or natural variability in bone formation. This led to an overall decrease in the number of calcified vertebrae at 6 dpf, but an increased trend in the rate of vertebrae calcification on average in *foxl1* mutants in comparison with WT siblings at 10 dpf (Figure 5B,C). The average number of vertebrates calcified (based on primary centre of ossification) for WT and *foxl1^-/-^* was 5 and 4 approximately by 6 dpf (with no change in body length; Supplemental Figure S3) and 8 and 10 approximately by 10 dpf (Figure 5B,C).

**Figure 6:**
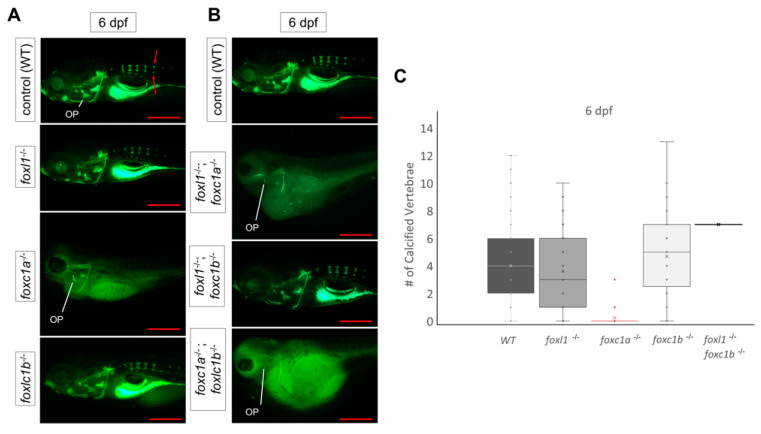
Calcein staining illustrating the impact of *foxl1, foxc1a*, and *foxc1b* lone and combined loss on axial skeleton development and calcification. (**A**) WT, *foxl1^-/-^*, *foxc1a^-/-^*, and *foxc1b^-/-^* embryos at 6 dpf showing normal formation of the vertebrae in *foxl1* and *foxc1b* mutants. *foxc1a^-/-^* embryos did not develop or calcify vertebrae and had large abdomen cavity edemas that grew over time. (**B**) WT; *foxc1a^-/-^; foxl1^-/-^, foxc1b^-/-^; foxl1^-/-^,* and *foxc1a^-/-^; foxc1b^-/-^* embryos at 6 dpf. Double *foxc1a^-/-^; foxl1^-/-^* embryos exhibit larger edemas and a lack of most calcified structures at 6 dpf, including the operculum (OP). *foxc1b^-/-^; foxl1^-/-^* embryos had no significant change. *foxc1a^-/-^; foxc1b^-/-^* mutants elicited similar results as *foxc1a^-/-^; foxl1^-/-^* embryos, including edemas and lack of craniofacial calcification. (**C**) Boxplots illustrating the variability in the number of vertebrae calcified in *foxl1^-/-^, foxc1a^-/-^*, *foxc1b^-/-^*,and *foxl1^-/-^; foxc1b^-/-^* mutants in comparison with WT embryos at 6 dpf. Red arrows indicate primary centres of ossification within the vertebrae. Scale bars are 500 µm.

*foxc1a* mutants lack any axial skeleton formation at 6 dpf while presenting with edemas around the heart and body cavity that grew over the course of time (Figure 6A), thus preventing the analysis of spine calcification in *foxl1^-/-^, foxc1a^-/-^* mutants entirely (Figure 6B). It should be noted, however, that the lateral views presented in Figure 6 reiterate the increased severity of craniofacial phenotypes observed in *foxl1^-/-^, foxc1a^-/-^* mutants, including decreased calcification of the operculum previously shown in in Figure 4. *foxc1b* mutant embryos did not exhibit any abnormal phenotypes regarding axial skeletal development at 6 dpf (Figure 6A), nor did double *foxl1^-/-^, foxc1b^-/-^* mutants (Figure 6B). 

To further investigate the calcification of the zebrafish skeleton, dual-energy X-ray absorptiometry (DXA) was performed on *foxl1* mutants to measure bone mineral density as per Green et al. (Supplemental Figure S2) [70]. No significant difference was found between WT siblings and heterozygous or homozygous *foxl1* mutants alone at 5.5 months old (Supplemental Figure S2A). As both *foxl1* and *foxc1b* have been associated with osteoporosis, we also assessed *foxl1^+/-^; foxc1b^+/-^* double heterozygous mutants and WT controls at 1 year of age, and similarly, no difference in their BMD was observed (Supplemental Figure S2B). However, our *foxl1* homozygous mutants exhibited wider heads and jaws along with longer jaws in comparison to their WT counterparts (Supplemental Figure S3A,B). 

### 3.6. Expression of Genes Required for Bone Formation in Forkhead Mutants

**Figure 7:**
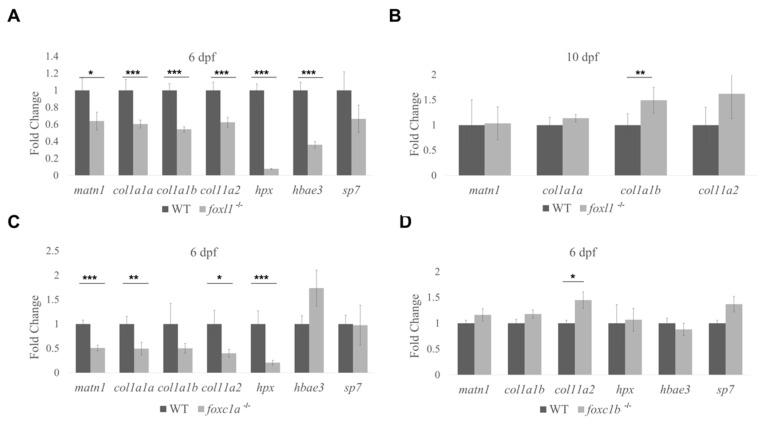
Differential gene expression in forkhead mutants. *foxl1^-/-^* (**A**), *foxc1a^-/-^* (**C**), and *foxc1b^-/-^* (**D**) embryos at 6 dpf using TaqMan RT-qPCR to confirm genes of interest from RNA-Seq screen. Targets of interest were examined at 10 dpf in *foxl1* mutants (**B**), demonstrating a recovery in gene expression. Fold change between WT and *foxl1^-/-^* plotted with WT values set to 1 (* *p* < 0.05, ** *p* < 0.01, *** *p* < 0.001).

To further demonstrate the role of *foxl1* on skeletal development, we assessed the expression of genes with known roles in cartilage and bone formation in zebrafish *foxl1*, *foxc1a,* and *foxc1b* mutants. To determine candidate target genes of *foxl1,* an RNA-Seq screen was completed on WT and *foxl1*^-/-^ embryos at 6 dpf. Prominent genes with differential expression were then validated using TaqMan Real-Time quantitative PCR (RT-qPCR). At 6 dpf, we identified markers of cartilage and heme-binding proteins as differentially expressed (Figure 7). Chondrocyte markers such as *matrilin* 1 (*matn1,* 36% reduction), *collagen type 1 alpha 1a* (*col1a1a*, 40% reduction), *collagen type 1 alpha 1b* (*col1a1b*, 46% reduction), and *collagen type 11 alpha 2* (*col11a2*, 38% reduction) saw statistically significant (*p* < 0.05) reductions in gene expression based on total whole embryo RNA (Figure 7A). *hemoglobin alpha embryonic-3* (*hbae3*, 64% reduction) and *hemopexin* (*hpx*, 92% reduction), both important factors in heme-binding and transport, were even more so reduced in *foxl1* mutants. *osterix (sp7),* a marker of osteoblast differentiation, was downregulated in *foxl1* mutants but did not reach statistical significance (Figure 7A). These data demonstrate the reduction in genes required for collagen and bone formation at 6 dpf, consistent with the delay in chondrogenesis and subsequent calcification of aspects of the craniofacial skeleton at this developmental timepoint. To further illustrate the genetic redundancy that may allow overtly normal jaw development in the absence of *foxl1,* we tested the expression of the same genes in *foxc1a* and *foxc1b* mutants. Much like *foxl1* mutants, *foxc1a*^-/-^ embryos saw significant reduction in *matrilin* 1, *collagen type 1 alpha 1a, collagen type 11 alpha 2,* and *hemopexin*, indicating target gene overlap (Figure 7C). Intriguingly *foxc1b^-/-^* embryos saw an increase in *collagen type 11 alpha 2* (Figure 7C,D), suggesting it acts as a repressive regulator in comparison to the enhancing mechanism of *foxl1* and *foxc1a*. 

As a recovery was observed in the formation of craniofacial cartilages and bone in *foxl1*^-/-^ embryos by 10 dpf, the gene expression of *matrilin* 1, *collagen type 1 alpha 1a,* and *collagen type 11 alpha 2* were assessed to determine the source of recovery. All genes saw a return to WT level gene expression, notably with *col1a1b* significantly overexpressed in direct contrast to its previous reduction at 6 dpf (Figure 7B). This indicates that despite *foxl1* loss, increased gene expression of necessary factors is upregulated by other means to achieve a largely normal skeleton throughout the maturation of the zebrafish larvae. 

## 4. Discussion

Here, we report on a *foxl1* mutant strain in zebrafish as a potential model of otosclerosis and osteoporosis. Structures in the mammalian middle ear are homologous with the ceratohyal, palatoquadrate, and hyomandibular jaw bones in zebrafish [48,51,52,53,71] and as such, the malformation of these bones leads to the loss of sound conduction in mammals and jaw formation in zebrafish. Despite abundant *foxl1* expression in the pharyngeal arches and a delay in cartilage formation and calcification in the jaw, loss of *foxl1* does not significantly affect the overall structure of the craniofacial skeleton, consistent with recent reports using strain harboring a *foxl1* nonsense mutation [25]. In agreement with a delay in cartilage formation and calcification at 6 dpf, we do observe a reduction in the expression of cartilage markers such as *matrillin1 and col1a1a/b* in *foxl1* homozygous mutants at this developmental time point. In accordance with the partial phenotypic recovery seen by 10 dpf, we also see a recovery in gene expression. Conversely, previous *foxl1* morpholino studies have shown much more pronounced phenotypes including abnormalities in craniofacial skeleton, midbrain, eye, and pectoral fin [72]. While these discrepancies may be due to morpholino off-target effects, genetic compensation in CRISPR-generated INDEL mutants [73,74,75] may also play a role. 

While *FOXL1* (and *FOXC2*) have been associated with osteoporosis in humans, and the co-occurrence of otosclerosis and osteoporosis has been reported [65,66,76], we did not find any defects in development of the larval zebrafish axial skeleton in *foxl1* or *foxc1b* (*FOXC2 homolog*) mutants. The axial skeleton in *foxl1^-/-^* mutants showed an increased trend of vertebrae calcified by 10 dpf but remained largely unaffected by the loss of *foxl1*. It is also possible that *foxl1* may play a specific role in endochondral ossification, which includes the jaw, but may not regulate intramembranous bone development that occurs in the zebrafish spine. No differences in BMD are observed in adult *foxl1* mutant zebrafish using DXA scanning; however, detection of BMD in fish using this method may not be sensitive enough to determine small changes in BMD, and only relatively young fish (5.5 months of age) were tested in this study. Additional studies assessing BMD in varying ages of adult fish through more sensitive µCT imaging could determine whether *foxl1* mutants have defects in maintaining BMD as they age.

Since *foxl1* has been shown to be expressed in cartilage and bone-specific tissues and is associated with bone remodelling diseases such as otosclerosis and osteoporosis [35,36,57], it is interesting that the loss of *foxl1* does not cause such profound bone phenotypes in the head or axial skeleton in the developing zebrafish. This indicates that, as with many other FOX genes, *foxl1* may only act as minor genetic modifier in development [28,72,77,78]. Our data indicate that when mutated alone, the development of bone and cartilage is predominantly normal in zebrafish larvae, which only exhibit minor delays by 10 dpf. However, when mutated in conjunction with *foxc1a*, another FOX gene known to be central in upper craniofacial cartilage formation and somite patterning, further loss of bone is observed than in *foxc1a* mutants alone. In zebrafish, bones in the craniofacial skeleton of *foxc1a* mutants that began to calcify, such as the operculum, did not form or were even more malformed in *foxl1^-/-^; foxc1a^-/-^* mutants. Taken together with qPCR data indicating that *foxl1* shares similar targets as *foxc1a*, the results support a model whereby *foxl1* may act as a modifier locus for *foxc1a*. Alternatively, the increased severity of phenotypes through the combined loss of *foxl1* and *foxc1a* may represent reduced growth and failure to thrive when compared to *foxc1a* mutants alone, as opposed to a specific effect on bone development. This possibility should be explored further. Heterozygous loss of *FOXC1* in humans results in Axenfeld–Rieger syndrome (ARS), which results in increased risk for glaucoma and variable systemic anomalies including defects in craniofacial bone development [79]. Recently, an atypical *FOXC1*-attributable ARS patient was described with clinical otosclerosis [33], again highlighting the potential overlapping functions of *foxl1* and *foxc1a*. 

Another novel hypothesis indicating *foxl1’s* possible involvement in bone development and maintenance is its regulation of *hemopexin*, which is responsible for high-affinity heme-binding and transport to the liver for degradation, preventing oxidative stress and iron loss [80,81] as well as *hemoglobin alpha embryonic-3* that is involved up-stream of the oxygen transport chain as an early marker of erythrocyte development [82]. Both genes were significantly downregulated in *foxl1*^-/-^ mutants, and there is a known association between heme-transport/blood diseases such as anemia and low BMD leading to fracture over time [83,84,85]. This could suggest that a loss of *foxl1* may cause an increase in oxidative stress, which in turn may exacerbate the mild cartilage and bone phenotypes observed during growth and remodelling. This hypothesis warrants further investigation. 

## 5. Conclusions

In zebrafish, *foxl1* is required for the expression of key collagen genes but plays only a minor role in the formation and maintenance of zebrafish jaw cartilages and bones. Given the homology of zebrafish jawbones and mammalian middle ear bones, *foxl1* mutant zebrafish serve as a useful model to gain an understanding of the mechanistic insights regarding the pathophysiology of otosclerosis. Additional analyses will be needed to determine if BMD defects occur in *foxl1* mutant zebrafish and whether they may serve as a useful model of osteoporosis. Our study supports the hypothesis that *foxl1* and *foxc1a* function together to regulate bone development; however, the manner in which *foxl1* and *foxc1a* function together to achieve optimal expression of cartilage and bone markers has yet to be determined and needs further exploration. This study highlights a new *foxl1* mutant zebrafish line, finding novel target genes of *foxl1* that could be utilized for new therapies to treat otosclerosis. 

## Data Availability

All available raw data are available from the Principal Investigator, Cutis R. French.

## Supplementary Materials

The following supporting information can be downloaded at: https://www.mdpi.com/article/10.3390/genes13071107/s1, Table S1: Primer and sgRNA sequences used in this study, Figure S1: CRISPR-induced mutation of *foxl* in zebrafish, Figure S2: Loss of *foxl1* does not affect bone density in adult zebrafish, Figure S3: Loss of *foxl1* results in increased head and jaw width, and jaw length, but does not affect body length.
