# Supplementary material for: Mutation of foxl1 Results in Reduced Cartilage Markers in a Zebrafish Model of Otosclerosis
Source: Genes (Basel). 2022 Jun 21;13(7):1107. doi: 10.3390/genes13071107 (PMC9319681; doi:10.3390/genes13071107)
Supplement: Supplementary file 1 [file genes-13-01107-s001.zip › genes-1690325-supplementary.pdf]

Supplementary Materials

Table S1. Primer and sgRNA sequences used in this study.

Sequence (5′-3′)	Purpose
GTTTTGGAGAGCAGTCATGCAG	*foxc1a* probe, forward primer
GCGTTGGAGGTAGTCGAGATAG*	*foxc1a* probe, reverse primer
TGAAGCAAAGGGAGGAAGAGAC	*foxc1b* probe, forward primer
ATAGAGGAGGCGTTTGTTGTGT*	*foxc1b* probe, reverse primer
GTCTCTCCGCTCAAGCAAAATC	*foxl1* probe, forward primer
CGTAGGAGAGGACAGGAACAAC*	*foxl1* probe, reverse primer
AGGCATCATCCCCGCGCT	*foxl1* gRNA sequence 1
AGCGCGGGGATGATGCCT	*foxl1* gRNA sequence 2

*A T7 promoter sequence (TAATACGACTCACTATAGGG) was added to the 5′ end of the reverse primer to generate antisense probes.

A

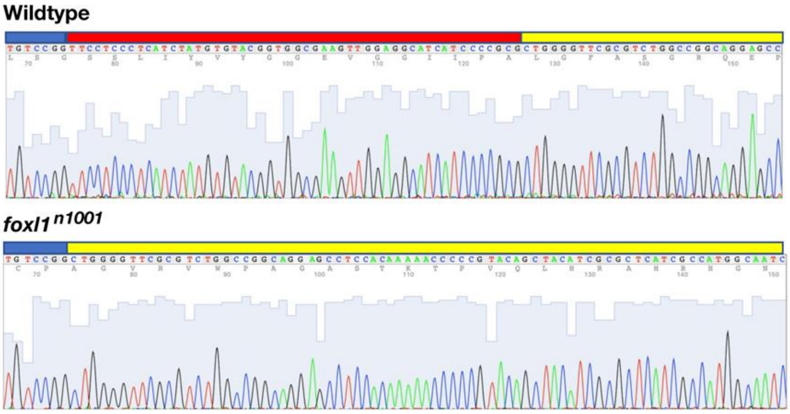

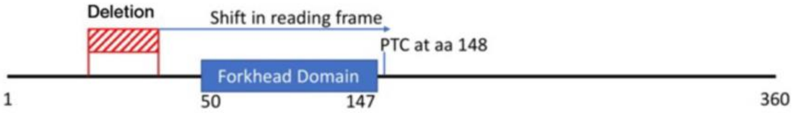

MSLYHSPVPQSGVAASALGLSGWGSRLAGRSLHKNPRTATSRSSPWQSRTRRTSARPSAGSISSSWTASHIIT
TTNKVGRTQSAITSRSTTVSLKCPERRGDRAKAATGRWTPNAWTCLRTETTGGGRESAGLRTQVTPKSVTRE
LG

B

```
HUMANfoxl1          MSHLF--------DPRLPALAASPMLYLYGPERPGLPLAFAPAAALAASGRAETPQKPPY      52
ZEBRAFISHfoxl1      MSLYHSPVPQSGVAASALGLSGSSLIYVYGGEVGGIIPAL-----GFASGRQEPPQKPPY      55
                    **  .              .*:.* ::*:** *  *:  *:       **** * ******

HUMANfoxl1          SYIALIAMAIQDAPEQRVTLNGIYQFIMDRFPFYHDNRQGWQNSIRHNLSLNDCFVKVPR      112
ZEBRAFISHfoxl1      SYIALIAMAIKNAPDKRATLSGIYQFIMDRFPYYHDNKQGWQNSIRHNLSLNDCFIKVPR      115
                    **********::**::*.**.***********:****:*****************:****

HUMANfoxl1          EKGRPGKGSYWTLDPRCLDMFENGNYRRRKRKPKPGPGAP---EAKRPRAETHQRSAEAQ      169
ZEBRAFISHfoxl1      EKGRPGKGSYWTLDTKCLDMFENGNYRRRKRKCRTQDTGDTKVGHKRTRVTSFKLHQGAQ      175
                    ************** :**************** :     .       ** *. :.:    **

HUMANfoxl1          PEAGSG-AGGSGPAISRLQAAPAGPSPLLDGPSPPAPLHWP-----------GTASPNED      217
ZEBRAFISHfoxl1      SEKVSPLKQNPGRDIKEKS-NDTQPQNEEENVASESAKDWCLASSTTIVSLPRCTTPERS      234
                     *  *    . *  *.. .    : *.   :. :  :  .*                ::*:..

HUMANfoxl1          AGDAAQ------GAAAVAVGQAARTGDGPGSPLRPASRSSPKSSDKSKSFSIDSILAGKQ      271
ZEBRAFISHfoxl1      STVAVNTHTPLSSASETRVPVKSDTGRAQSGDAKSKESNPRKTTDKAKEFSIDSILSKKE      294
                    :  *.:       .*: . *   : ** . ..  :  . .  *::**:*.*******: *:

HUMANfoxl1          GQKPPSGDELLGGAKPGPGGRLGASLLAASSSLRPPFNASLMLDPHVQGGFYQLGIPFLS      331
ZEBRAFISHfoxl1      NQFQRRCAAA-GGASVDSR---------------GYALASSLIAHAHPQLYPRGFPLCS      337
                    .*         ***. .                   :  :  *  *.:  :*  *:*: *

HUMANfoxl1          YFPLQVPDTVLHFQ---------      345
ZEBRAFISHfoxl1      YLSLTCPDKILSLTEKTDEKLFF      360
                    *: *  **.:* :
```

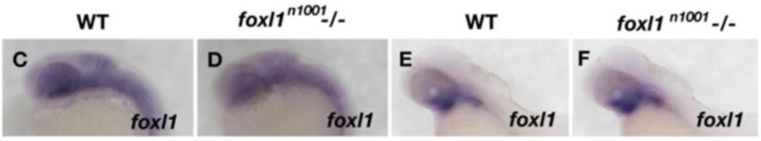

Figure S1. CRISPR induced mutation of *foxl* in zebrafish. A 52 bp deletion prior to the forkhead DNA binding domain, denoted as *foxl1*^{n1001}. Sequenced denoted by the red box is present in wildtype, but not *foxl1*^{n1001} mutants (A). This causes a shift in the open reading frame leading to a predicted premature termination codon at amino acid 148. The predicted protein would contain only 22 amino acids (black text) normally found in the Foxl1 protein. Protein alignment of zebrafish and human FOXL1 is presented in (B). Expression of *foxl1* is not affected in homozygous mutants at 24 hpf (C and D) and 48 hpf (E and F), indicating that as expected, the mutant allele is not targeted for degradation through the nonsense mediated decay pathway.

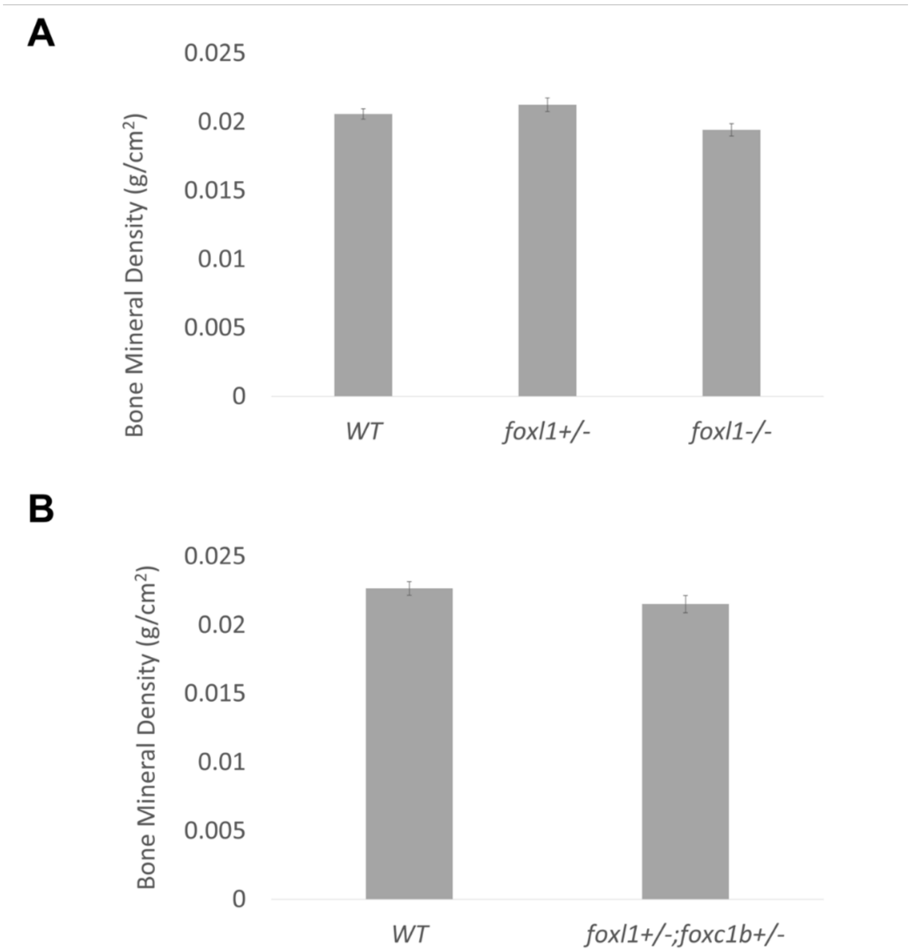

Figure S2. Loss of *foxl1* does not affect bone density in adult zebrafish. Analysis of *foxl1* mutants at 6 months of age does not reveal any bone mineral density defects in heterozygous or homozygous fish (A). At one year of age, fish heterozygous for both *foxl1* and *foxc1b* mutations does not have any defects in bone mineral density (B).

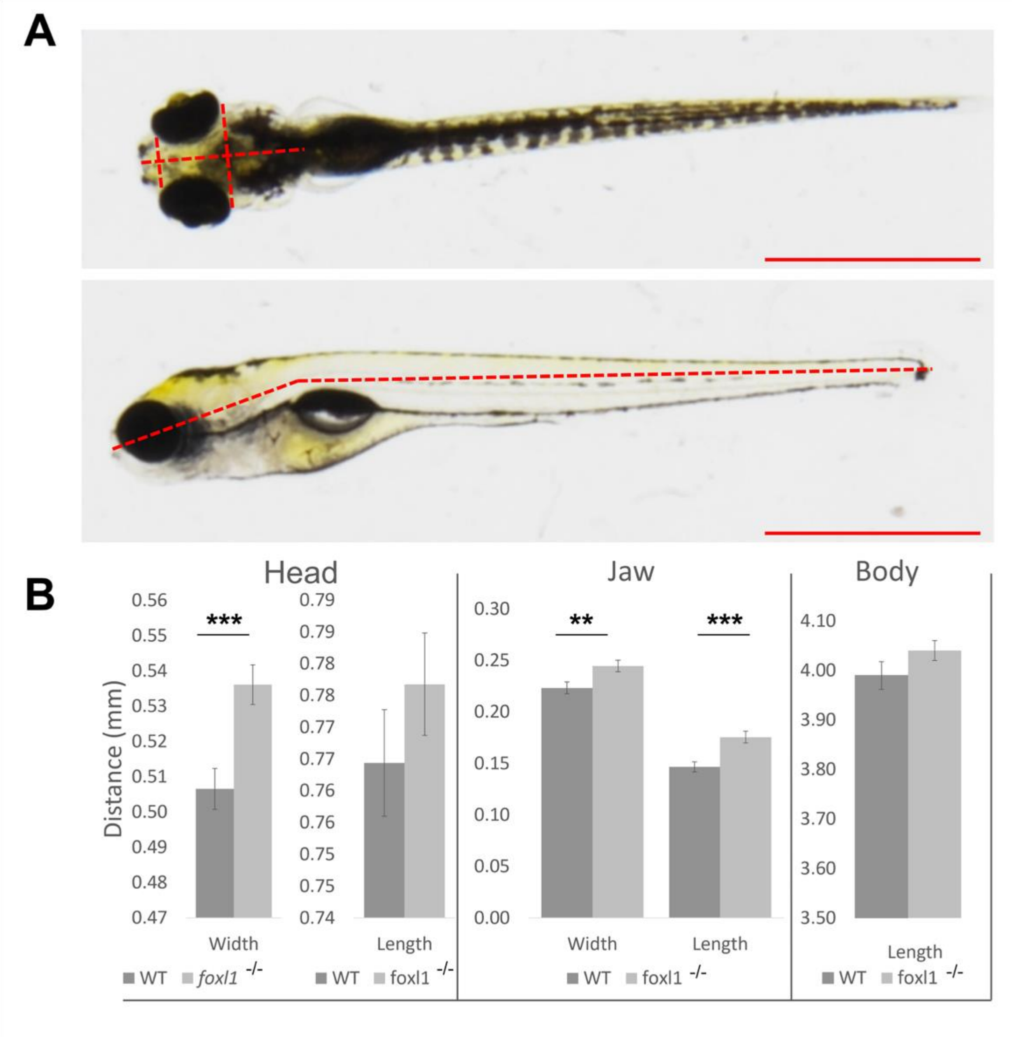

Figure S3. Loss of *foxl1* results in increased head and jaw width, and jaw length, but does not affect total body length at 6 dpf. (A) WT embryo indicating the axes measured (red, dotted lines). Jaw width was measured at the widest point of the palatoquadrate, with length being measured from the most distal end of the mouth to the end of the palatoquadrate bone. Head width was measured at its widest point immediately posterior of the eyes, while length was determined from the most distal end of the mouth to the most anterior portion of the Weberian apparatus. Body length was determined by measuring along the lateral line and middle of head. (B) *foxl1* (n = 28) mutants exhibit both wider heads (p = 000089) and jaws (p = 0.01254), along with longer jaws (p = 0.00063) in comparison to WT siblings (n = 24). No difference was observed in head or total body length. Significance was determined using a two-tailed T-test. Scale bars are 1 mm.
